# Single-incision laparoscopic cholecystectomy reduced postoperative pain than three-incision laparoscopic cholecystectomy in patients with large gallstone, a retrospective study

**DOI:** 10.3389/fsurg.2024.1448684

**Published:** 2024-12-05

**Authors:** Zhiheng Zhang, Jiawei Xu, Decai Yu, Nacheng Lin, Jin Peng

**Affiliations:** ^1^ Division of Hepatobiliary and Transplantation Surgery, Department of General Surgery, Nanjing Drum Tower Hospital, Affiliated Hospital of Medical School, Nanjing University, Nanjing, China; ^2^ Department of Obstetrics and Gynecology, Nanjing Drum Tower Hospital, Nanjing, Jiangsu, China

**Keywords:** single-incision laparoscopic cholecystectomy, gallstone, gallstone size, postoperative pain, mini invasive

## Abstract

**Aim:**

To compare the short-term outcomes between SILC and TILC depending on gallstone size.

**Material and methods:**

Data from 114 patients with gallstones who underwent cholecystectomy hospitalized in Nanjing Drum Tower Hospital between June 2022 and October 2023 were collected. The gallstone diameter, the operation time, estimated blood loss, post-operative pain, complications post-operation, and length of hospital stay were all collected and examined.

**Results:**

Of the 114 patients included in this study, 61 underwent SILC, and 53 underwent TILC. The pain score 6 h, 24 h post-operation was higher in the TILC group compared with the SILC group. Patients were divided into large (diameter > 2 cm) and small groups (diameter < 2 cm), larger gallstones significantly increased operation duration in the SILC group. For the TILC group, large gallstones significantly increased blood loss during the operation. The blood loss and pain scores were higher in the TILC group compared with the SILC group for patients with large gallstones.

**Conclusion:**

In this study, SILC and TILC both had comparable postoperative outcomes, while SILC significantly reduced postoperative pain than TILC. Moreover, SILC might be a suitable option for patients with larger gallstones (diameter > 2 cm) and helps reduce blood loss and postoperative pain.

## Introduction

Gallbladder disease is one of the most common and costly diseases in the world (1, 2). With the improvement of surgical techniques and laparoscopic instruments, laparoscopic cholecystectomy (LC) has been identified as the gold standard for gallbladder resection, especially for gallstones (3). The size of a gallstone always has a higher risk of complications and higher technical difficulties during LC (4, 5).

Three-incision laparoscopic cholecystectomy (TILC) is widely applied worldwide for benign gallbladder disease. In 1997, Navarra et al. performed the first single-incision laparoscopic cholecystectomy (SILC) by using two trocars through one sub-umbilical incision (6). After that, the application of SILC was generally accepted in clinical practice. Several groups have compared the safety and efficiency of SILC with TILC (7, 8), SILC has reduced postoperative pain, shorter hospitalization duration, reduced port-site complications, better cosmesis, and a higher complication rate compared with TILC (7, 8). However, no study investigates the impact of gallstone size on the outcome of SILC or TILC in the treatment of gallstones. The purpose of this study was to compare the short-term outcomes between SILC and TILC depending on gallstone size.

## Material and methods

### Patients

This retrospective study, conducted at Nanjing Drum tower hospital, analyzed the records of 114 consecutive patients with gallstones. Inclusive criteria: patients with gallbladder stones who underwent cholecystectomy from June 2022 to October 2023 in the ambulatory surgical administration center of Nanjing Drum Tower Hospital. Exclusive criteria: those with AC, abnormal liver function blood test, an endoscopic retrograde cholangiopancreatography (ERCP) before operation, an endoscopic ultrasound (EUS) before operation, and those undergoing combined surgeries were excluded from the study. Patients without magnetic resonance cholangiopancreatography (MRCP) were excluded. SILC was performed according to the patient's choice owing to better cosmetic requirements. TILC was chosen based on the surgeon's decision according to the patient's conditions and the availability of surgical instruments.

### Operation procedures

The operation was performed by a group of experienced surgeons in the Hepatobiliary surgery department of Nanjing Drum Tower Hospital. The patient was rotated 10–15° to the right upward axis while supine in the reverse Trendelenburg position during SILC. Following the skin incision, the umbilical scar was separated from the fascia, allowing for greater flexibility when the ports were introduced. CO_2_ insufflation produced a pneumoperitoneum of 12 mmHg. Next, via the umbilical incision, a single clear Glove port is introduced. A critical perspective on safety was examined throughout the procedure. An initial step was the ligation of the cystic artery and cystic duct with a 5- or 10-mm Hem-O-Lok clip (Mindray). Using monopolar equipment (Mindray), the gallbladder was removed from the liver bed and placed into a specimen bag. In patients who were at high risk of bleeding or showed signs of a bile leak, a latex tube drain was placed. Interrupted sutures were used to seal the fascia. TILC was performed with the patient supine and three trocars [one each on the umbilicus (10 mm), subxiphoid (12 mm), and right upper quadrant (5 mm)]. Cholecystectomy was conducted in the same way as previously described, with the specimen recovered through the subxiphoid using a specimen bag.

### Assessed factors

In this study, the gallstone diameter, operation duration, estimated blood loss, post-operative pain, complications post-operation, and length of hospital stay were all examined. All patients had a unified postoperative control scheme as mentioned below. Surgical postoperative complications were defined as bile duct injury, bile duct stone, postoperative bleeding, and abdominal infection. Operation time and blood loss were obtained from surgical records.

### The management and outcome of pain

A senior pharmacist (Dr Yao Du), Dr Jiawei Xu, and two senior nurses (Ms Jie Hu, and Ms Liping Zhou) in our center were responsible for patients' pain recording and administration. The nurses educated patients about their pain pre-operation and administered their medication at the right timing and dosage according to the dynamic changes in the patient's pain scores (9). The numerical rating scale (NRS) for pain is a scale for pain in which 0 represents “no pain” and 10 represents “unbearable pain” (8). All patients were asked to assign a number to refer to their average pain at 0, 6, and 24 h after surgery. All patients had a unified postoperative control scheme. All patients were directly intravenously administered flurbiprofen axetil (50 mg in 100 ml of 0.9% saline) after the operation. When the pain score on the NRS (10) was 4 or higher, additional flurbiprofen axetil injection could be administered intravenously. In case the flurbiprofen axetil was contraindicated, patients received propacetamol 50 mg intravenously. The dosage of the pain was counted.

### Statistical analysis

A student's *t*-test or an analysis of variance was used to compare the means and standard deviations (SDs) of continuous variables. A chi-square test was used to compare categorical variables, all of which were two-sided, and a *P*-value of less than 0.05 was considered statistically significant. Multivariable analysis was performed using logistic regression analysis. The analyses were conducted using GraphPad Prism 9.0 or SPSS Statistics.

## Results

**Figure 1:**
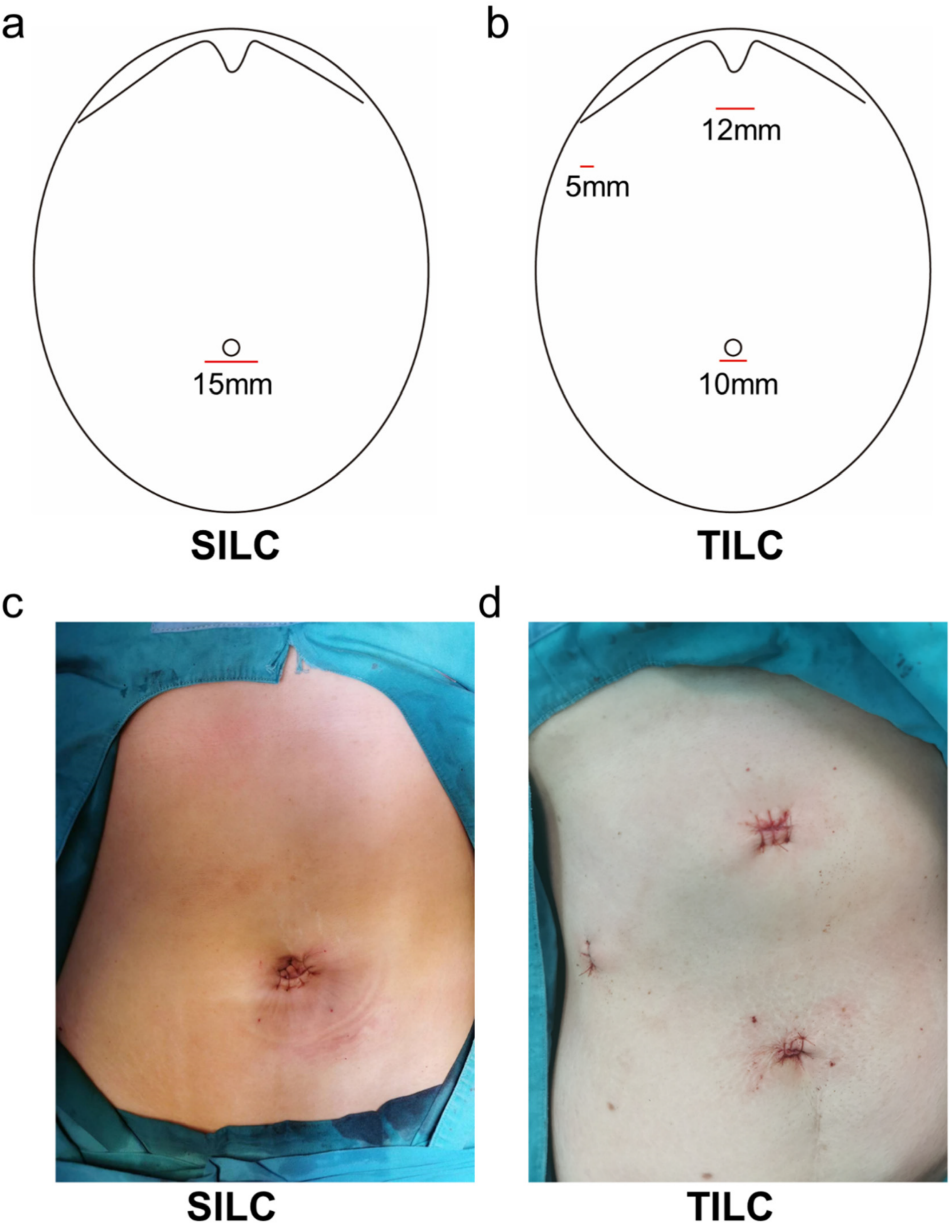
Illustration of the incision site. **(a,b)** The diagram show the position of incision site of SILC and TILC. **(c)** The picture shows the incision site of the SILC. **(d)** The picture shows the incision site of TILC.

**Figure 2:**
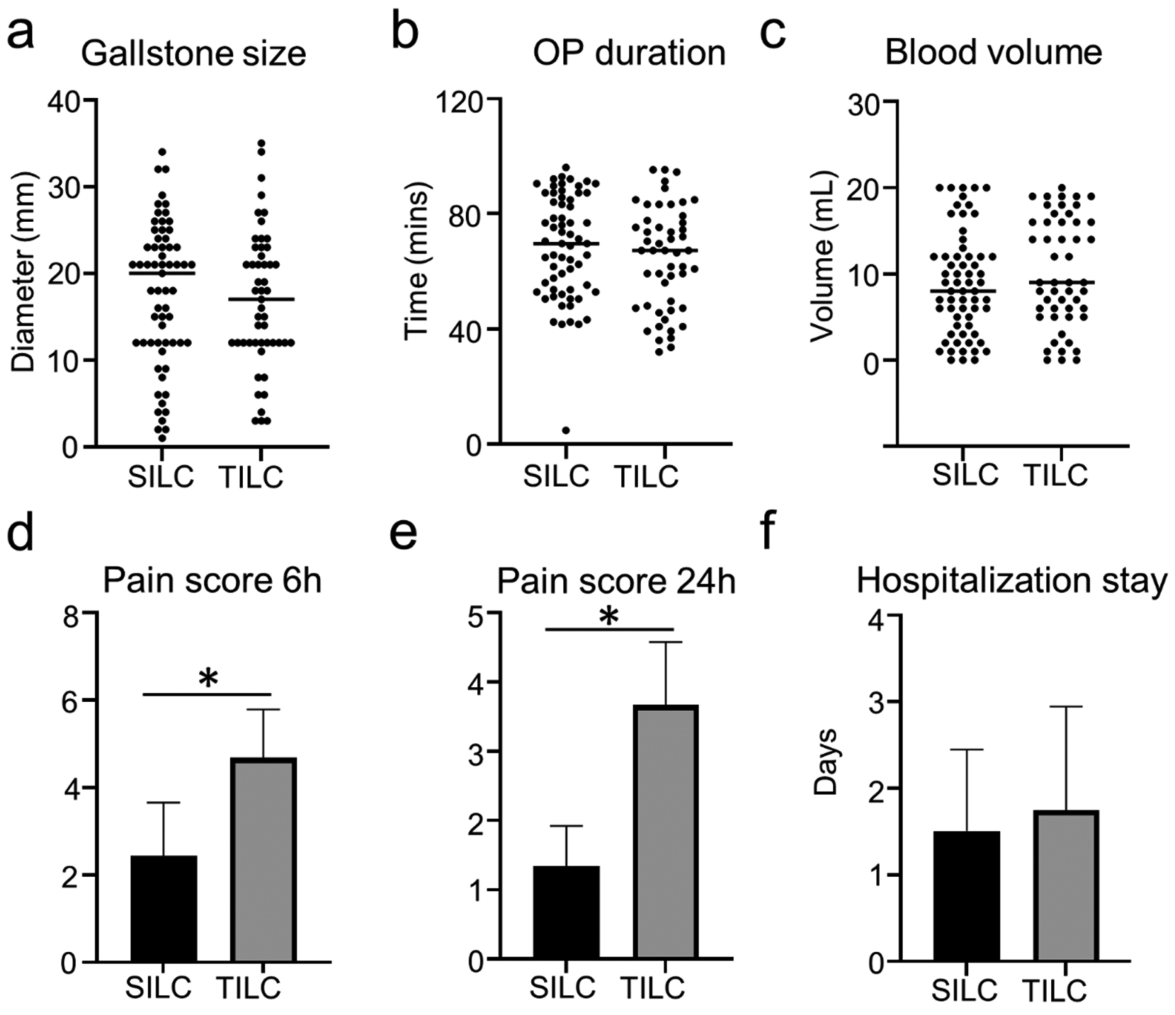
Patients in the SILC group showed decreased post-operative pain compared with patients in the TILC group. **(a)** The diameter of gallstones shows no difference in the two groups. **(b)** The plot chart shows no difference in operation time between the SILC and TILC groups **(c)**. The plot chart shows no difference in the blood volume between the SILC and TILC groups. **(d)** The bar chart shows the decreased pain score 6 h post-operation in SILC compared with the TILC group. **(e)** The bar chart shows the decreased pain score 24 h post-operation in the SILC group compared with the TILC group. **(f)** The bar chart shows no difference in the hospitalization stay between the SILC and TILC groups. *:*P* < 0.05.

114 patients were included in this retrospective study, 63 underwent SILC, and 51 underwent TILC. Characteristics of both groups about gender, age, BMI, and length of disease are presented in Table 1. None of the differences in patient's baseline characteristics was significant between the two groups. A glove port with a snake liver retractor was used in SILC. A 15 mm infra-umbilical incision was made in SILC (Figures 1a,c). A 10 mm infra-umbilical incision, a 12 mm incision and a 5 mm incision were made in TILC (Figures 1b,d). Cholecystectomy was performed as usual. The gallstone size, operation duration, blood volume, and the length of hospitalization stay show no difference between SILC and TILC groups (Figures 2a–c,f). And pain score 6 h, 24 h post-operation was higher in TILC compared with SILC, 17 out of 63 SILC patients and 41 out of 51 TILC patients receiving a dose of pain killer 6 h post-operation (Figure 2d,e; Table 2). In conclusion, these results showed that SILC can significantly decrease patient pain score post-operation compared with that of TILC.

**Table 1:** Characteristics of patients.

Variable	SILC	TILC	*P*-value
Age (year)	48.21 ± 23/21	48.04 ± 17.32	0.953
Gender			
Male, *n* (%)	38, (60.13%)	26, (50.98%)	0.673
Female, *n* (%)	25, (38.87%)	25, (49.12%)	
BMI, mean m^2^/kg (SD)	26.54 ± 3.21	25.41 ± 2.15	0.324
Murphy sign	8, (12.69%)	7, (13.72%)	0.999
history of abdominal surgery	14, (22.22%)	7, (13.72%)	0.332
Complications			
Abdominal infection	2, (3.17%)	3, (5.88%)	0.407
Uncontrolled postoperative pain	0	0	–
Persistent nausea/vomiting	2, (3.17%)	4, (7.84%)	0.407

**Table 2:** Logistic regression analysis to identify the factors associated with post operation clinical outcomes in patients.

Variable	Univariate analysis	Multivariate analysis
	OR (95% CI)	OR (95% CI)
Age	0.758 (0.356–1.613)	
Gender	0.854 (0.723–1.349)	
Gallstone size	0.989 (0.945–1.036)	
OP duration	0.987 (0.970–1.005)	
Blood volume	1.037 (0.975–1.102)	
Pain score 6 h	4.631 (2.641–8.118)^b^	36.018 (8.869–114.269)^b^
Pain score 24 h	6.228 (2.132–10.239)^b^	12.343 (5.454–24.560)^b^
Hospitalization stay	0.984 (0.843–1.231)	

CI, confidence interval; OR, odds ratio.

*P* < 0.05.

^b^ *P* < 0.01.

**Figure 3:**
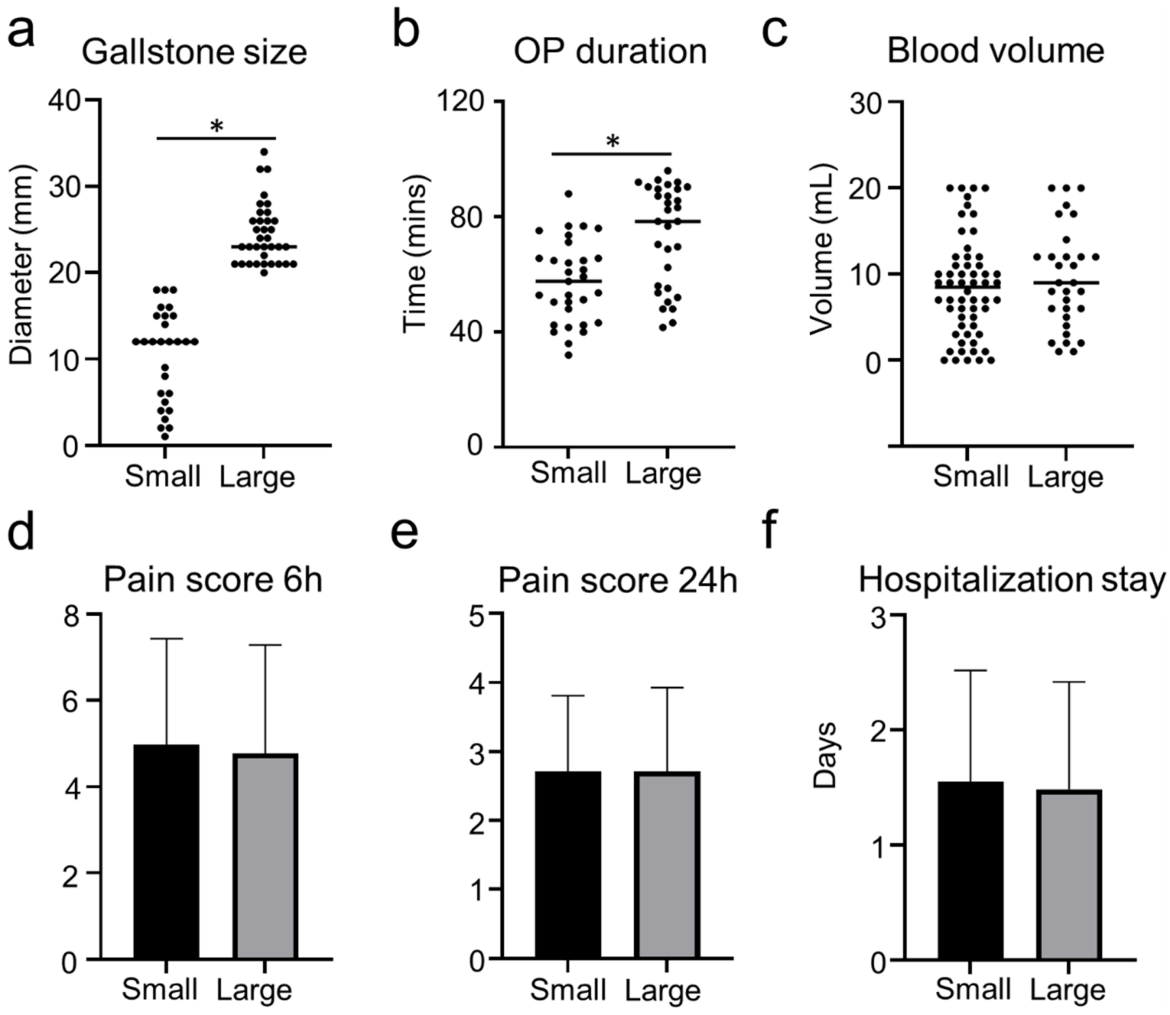
Outcome of patients who were receiving SILC with small gallbladder or large gallbladder. **(a)** The plot chart shows the diameter of gallstones. **(b)** The plot chart shows the increased operation time of patients who receive SILC with small gallbladders compared with patients with large gallstones. **(c)** The plot chart shows no difference in the blood volume between the two groups. **(d)** The bar chart shows no difference in the pain score 6 h post-operation between the two groups. **(e)** The bar chart shows no difference in the pain score 24 h post-operation between the two groups. **(f)** The bar chart shows no difference in the hospitalization stays between the two groups. *:*P* < 0.05.

**Figure 4:**
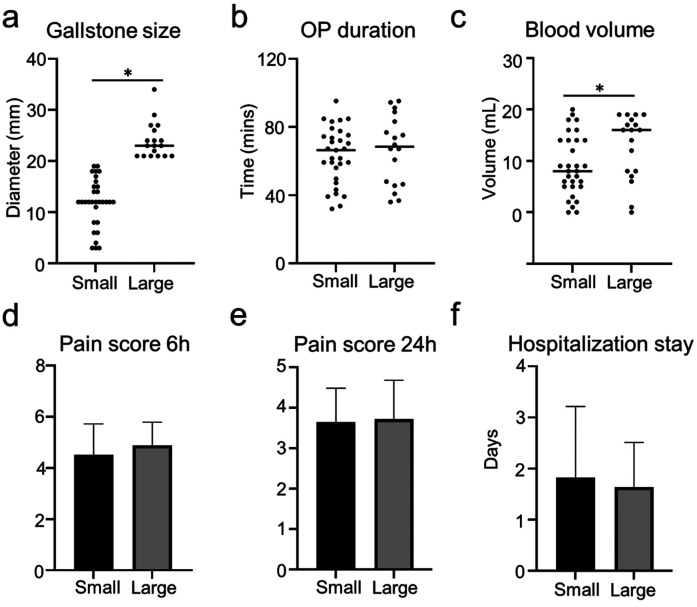
Outcome of patients who were receiving TILC with small gallstone or large gallstone. **(a)** The plot chart shows the diameter of gallstones. **(b)** The plot chart shows no difference in operation time between the patients who received TILC with small gallstones or large gallstones. **(c)** The plot chart shows the increased blood loss in the patients who received TILC with large gallstones when compared with patients with small gallstones. **(d)** The bar chart shows no difference in the pain score 6 h post-operation between the patients who received TILC with small gallstones or large gallstones. **(e)** The bar chart shows no difference in the pain score 24 h post-operation between the patients who received TILC with small gallstones or large gallstones. **(f)** The bar chart shows no difference in the hospitalization stay between the patients who received TILC with small gallstones or large gallstones. *:*P* < 0.05.

The size of the gallstone might impact the outcome of LC (4, 5). Thus, we investigate the effect of gallstone size on the clinical outcome of TILC and SILC. The diameters of the gallstones were evaluated and calculated through MRCP pictures. The medium number of the gallstone diameter was 2 [0.4–2.7] cm and was selected for the cutoff value. Patients were divided into small (gallstone diameter < 2 cm) and large (gallstone diameter ≥ 2 cm) groups according to the size of the gallstone (Figure 3a). Indeed, larger gallstones significantly increase operation duration in patients who receive SILC (Figure 3b). The blood volume, pain score, and hospitalization stay did not differ between the two groups (Figures 3c–f). For patients who receive TILC, patients were divided into two groups (Figure 4a), large gallstones significantly increased blood loss during operation (Figure 4c), while operative duration, pain score, and hospitalization stay did not differ between the two groups (Figures 4b,d–f).

**Figure 5:**
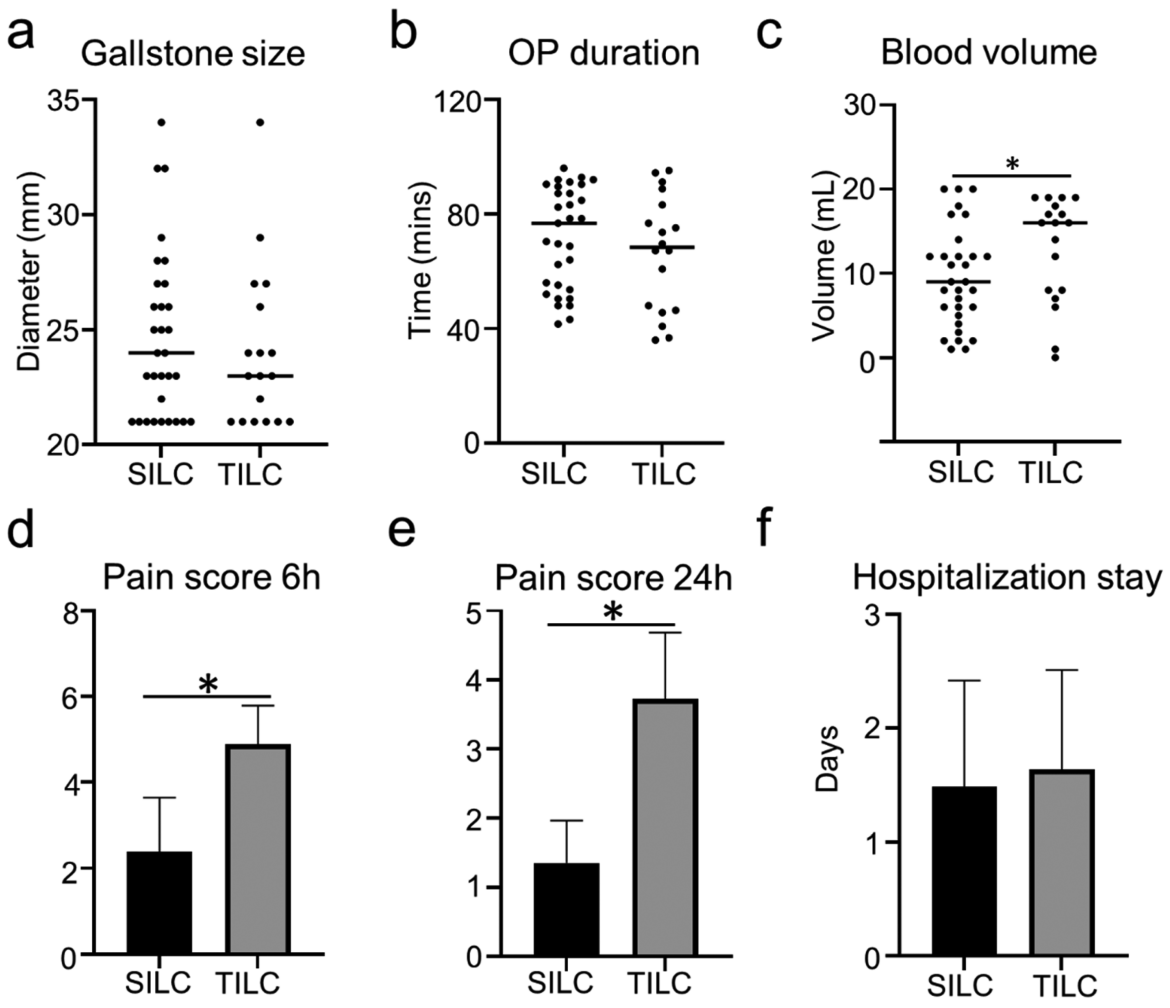
Outcome of patients with large gallstones who were receiving SILC vs TILC. **(a)** The plot chart shows the diameter of gallstones. **(b)** The plot chart shows no difference in the operation time between patients with large gallstones who were receiving SILC or TILC. **(c)** The plot chart shows increased blood loss in patients in the TILC group compared with patients in the SILC group. **(d)** The bar chart shows the increased pain score 6 h post-operation in patients in the TILC group compared with patients in the SILC group. **(e)** The bar chart shows the increased pain score 24 h post-operation in patients in the TILC group compared with patients in the SILC group. **(f)** The bar chart shows no difference in the hospitalization stay between patients with large gallstones who were receiving SILC or TILC. *:*P* < 0.05.

Thus, we analyzed the clinical outcome of TILC and SILC in patients with large gallstones. The size of the gallstones in the two groups showed no difference (Figure 5a). The operative duration and hospitalization stay did not differ between the two groups (Figures 5b,f). The blood volume and pain score were higher in the TILC group compared with the SILC group (Figures 5c,d,e).

## Discussion

Minimally invasive surgery, specifically LC, has become the preferred approach for treating gallbladder disease, particularly gallstones (3, 11, 12). SILC has emerged as a viable alternative to multiport LC, offering similar outcomes for gallbladder stone removal in cases without acute inflammation (12–15). This study aims to assess the impact of gallstone size on the effectiveness and safety of both SILC and TILC.

SILC is a minimally invasive surgical procedure specifically designed for gallbladder removal (6, 16, 17). Unlike traditional multiport LC, which requires multiple small incisions, SILC is performed through a single small incision, typically made in the patient's belly button (6, 13, 17, 18). The primary goal of this technique is to minimize visible scarring and potentially decrease postoperative pain levels (19, 20). In SILC, specialized instruments are inserted through the single incision, allowing the surgeon to visualize and remove the gallbladder. This approach offers the potential benefits of improved cosmetic outcomes and reduced postoperative discomfort (20, 21). Indeed, this study confirmed that SILC significantly reduced patient's post-operative pain compared with patients with TILC. This might be attributed to the only one incision was made. The decreased post-operative pain is essential for improving patient satisfaction, enhanced recovery, reduced postoperative complications (e.g., pneumonia, deep vein thrombosis, and delayed mobility), and decreased opioid consumption. Furthermore, previously published studies have shown that SILC and TILC did not differ in postoperative complications or hospital stays between the two groups (22, 23).

Contrary to previous studies (17, 24), the operation time in the SILC group was not longer than in the TILC group in the present study. There are several possible explanations for this observation. First, the varied experience and technical proficiency of the surgeons in this study might contribute to the result. Second, the complexity of the surgery, such anatomy of the gallbladder, the degree of inflammation, or the presence of complications can affect the operation time.

Generally, the patients with bigger gallstones is more difficult for the surgeons to remove the gallbladder and always with more complications (4). This study confirmed that larger gallstones significantly increased operation duration in patients who received SILC and more bleeding during the operation in patients who received TILC. Several factors might contribute to this result: the larger gallstones are always locked/located in the neck or Hartmann's pouch of the gallbladder, which leads to the increased difficulty of surgical procedures and exposing “critical view of safety”; The adhesions and inflammation caused by large gallstones also increased the difficulty of the surgery. Our study showed the increased difficulty of the operation in the treatment of patients with large gallstones.

This study had some limitations. First, this is a single-center retrospective study with a selective bias, and the patients were included from a single hospital, which made the sample size less comprehensive and precluded the establishment of causality. Second, only patients with gallstones were included, a large-scale study to include complications is needed in the future. Third, this study only conducts a short-term outcome, limiting the assessment of overall efficacy and safety.

Despite these limitations, SILC has similar postoperative outcomes while reducing postoperative pain as TILC. Moreover, SILC might be a good treatment option for patients with larger gallstones. Shortly, we plan to make a multicenter, large sample, prospective, controlled trial to confirm this result and minimize the bias in the study. And long-term outcomes will be conducted in the future.

## Data Availability

The original contributions presented in the study are included in the article/Supplementary Material, further inquiries can be directed to the corresponding authors.
